# Advances in the Management of Patients With Urothelial Carcinomas of the Bladder

**Published:** 2018-04-01

**Authors:** Amishi Shah, Emily Lemke

**Affiliations:** The University of Texas MD Anderson Cancer Center

## Abstract

For more than 30 years, there were no new treatments in bladder cancer. Then, in 2016, there were five drug approvals. JADPRO Live presenters delved into recent advancements and opportunities for improving outcomes for patients with bladder cancer.

Advances in treatment, including the successes achieved recently with immunotherapy, are improving outcomes for patients with bladder cancer, which is the sixth most common malignancy. At JADPRO Live 2017, this topic was discussed by Amishi Shah, MD, and Emily Lemke, DNP, AGPCNP-BC, AOCNP®, of The University of Texas MD Anderson Cancer Center.

"We had no new treatments in bladder cancer for 30 years, then last year we had five drug approvals," Dr. Shah said. "It’s an exciting time in bladder cancer."

Dr. Lemke introduced the discussion by describing the patient most often seen by advanced practitioners: an adult presenting with hematuria and perhaps other lower urinary tract symptoms who has been treated a few times for bladder infections that have not resolved. Further workup by the patient’s primary care physician or urologist reveals a mass, and the patient is referred to the oncologist.

The staging workup involves computed tomography (CT) of the chest, abdomen, and pelvis (with urogram), which evaluates for a thickening of the bladder wall; if thickening is shown, the patient moves on to cystoscopy. Depending on the symptoms, the patient may need brain magnetic resonance imaging (MRI) or bone scan. Patients with small cell histology also receive a brain MRI.

"The main differentiating factor in staging is muscle invasiveness," she said. This is staged T2 to T4aN0M0. Patients with stage T2 and higher are managed by oncologists, while Ta and T1 disease is managed by urologists (Figure 1).

**Figure 1:**
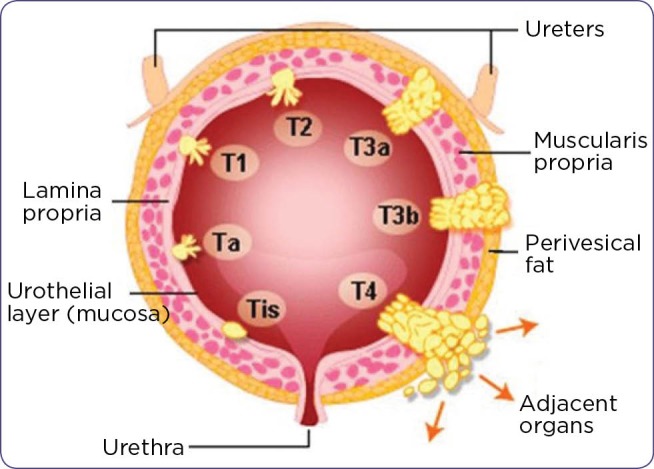
Bladder cancer staging (TNM). TNM = tumor, node, metastasis. Adapted from National Comprehensive Cancer Network (2017)

Once a bladder mass is identified, the patient with Ta and T1 disease undergoes transurethral resection of the bladder neck, followed by intravesicular therapy. Patients deemed at lower risk receive one course of intravesicular mitomycin, epirubicin, or gemcitabine; intermediate-risk patients receive chemotherapy for 1 year; high-risk patients receive intravesicular immunotherapy with Bacillus Calmette-Guérin.

Up-front cystectomy is recommended for patients with high-risk features, including multiple or large tumors, tumors of variant histology, concomitant carcinoma in situ in the bladder or prostatic urethra, or lymphovascular invasion.

"Patients with more advanced T1 disease or recurrence after intravesicular therapy may need up-front cystectomy," she added.

## MUSCLE-INVASIVE DISEASE

Dr. Shah described the approach to muscle-invasive disease. She noted that T2 tumors have encroached into the muscle layer, T3 tumors approach the perivesical fat, and T4 tumors have spread to adjacent organs.

T2 or greater disease warrants neoadjuvant chemotherapy, followed by surgical consolidation with dose-dense MVAC (methotrexate, vinblastine, doxorubicin [Adriamycin], cisplatin) or gemcitabine plus cisplatin (the gold standards). Early trials of neoadjuvant chemotherapy showed high rates of pathologic complete response as well as overall survival benefits. Compared with up-front cystectomy, neoadjuvant chemotherapy with MVAC increased survival from 46 to 77 months in one pivotal trial (Grossman et al., 2003).

"This seminal work led to the approval of neoadjuvant therapy, which became the gold standard," Dr. Shah said. Neoadjuvant chemotherapy is highly recommended for patients with signs of aggressive disease, such as variant histology (micropapillary, plasmacytoid, and others), hydronephrosis, lymphovascular invasion, or a palpable mass found with the patient evaluated under anesthesia.

"Neoadjuvant chemotherapy is key to successful treatment in these patients," she said. "But for patients with none of these risk factors, about 70% can be cured with surgery alone. We can often let them undergo up-front cystectomy."

"At our institution, if the patient has T2 disease just in the muscle, with no high-risk features, our long-term outcome is that 85% are cured with surgery alone. We feel a lot of these patients can be spared chemotherapy," she said, acknowledging that this approach is different from the more conservative standard treatment of T2 disease in which the practice is, "When in doubt, give neoadjuvant chemotherapy."

## STANDARD REGIMENS AND ALTERNATIVES

Two cisplatin-based regimens are the standard of care for treatment:

- Dose-dense MVAC: dosed every 2 weeks, this regimen reduces the time of treatment before surgery (8 weeks total treatment) and results in pathologic complete responses in up to 38% of patients; clinicians should make sure patients have no underlying cardiac problems before giving anthracycline
- Gemcitabine/cisplatin: dosed every 3 weeks (12 weeks total treatment) and results in pathologic complete responses in up to 26% of patients.

Other regimens can also be used, especially for the following conditions:

- Gemcitabine/paclitaxel (Taxol)/doxorubicin (GTA) for cisplatin-ineligible patients
- Etoposide/cisplatin (Platinol; EP) or alternating doublet ifosfamide/doxorubicin and etoposide/cisplatin (IA/EP) for patients with small cell histology (an aggressive variant)
- Cisplatin/gemcitabine/ifosfamide (CGI), dosed every 2 weeks using cisplatin dose of 50 mg/m²
- Ifosfamide/doxorubicin/gemcitabine (IA-Gem), dosed every 3 weeks by inpatient administration.

"This is our menu when we can’t use dose-dense MVAC or gemcitabine/cisplatin," Dr. Lemke said, although she emphasized the importance of cisplatin in this malignancy and urged practitioners, "If the patient can get cisplatin at all, you should try to give it."

"We’ve had good luck with GTA in patients with neuropathy, hearing problems, or renal dysfunction. We can also give CGI when patients have borderline creatinine clearance, or some tinnitus or neuropathy. For small cell histology, we’ve found EP to be efficacious, but it’s a tough regimen and these patients need to be fit. The same for IA-Gem—this is one of the big guns. It’s for very fit, young patients with few comorbidities, and we need to give this in the inpatient setting," she said.

## MONITORING AND SUPPORTIVE CARE

Patients receiving these regimens should be monitored for kidney function, electrolytes, hearing problems, neuropathy, cardiac problems, and cytopenias. "Check for toxicity in-between cycles when possible, as this gives you the opportunity to boost patients and keep them on schedule," she said.

This would include intravenous fluids, electrolyte repletion (especially potassium and magnesium), nausea/vomiting prophylaxis, and red blood cell infusions. Patients receiving neoadjuvant therapy are given pegfilgrastim.

## UPPER TRACT UROTHELIAL CARCINOMA

Upper tract urothelial carcinoma (UTUC) accounts for 5% of bladder cancers and "is its own entity, in a way," Dr. Shah said. For one thing, there is a hereditary predisposition, meaning that "automatically, Lynch syndrome should be on the radar," she said. "Any patient with UTUC should be tested for microsatellite instability (MSI) to screen for Lynch syndrome."

There are no clear guidelines on how to treat UTUC, and no well-defined role for neoadjuvant or adjuvant chemotherapy. Many patients undergo up-front cystectomy, but this is not always the optimal approach.

"If we see these patients from the beginning [i.e., they are not referred after surgery], we take a stance that is more like our treatment of bladder cancer. If there is any high-risk feature (such as high grade or sessile polyps), we recommend neoadjuvant chemotherapy before consolidative surgery, and cisplatin-based therapy is the gold standard," Dr. Shah said.

Because these patients are often MSI-high, there may be a role for immune checkpoint inhibitors, especially in patients who cannot receive cisplatin. The success rates approach 90% in this population. "Keep this in mind in UTUC patients," she said.

As in bladder cancer, neoadjuvant chemotherapy can be very beneficial in UTUC, as it can eradicate micrometastases, downsize tumor for surgery, reduce the risk of recurrence, and be tolerated better than adjuvant chemotherapy, she added.

## METASTATIC UROTHELIAL CANCER AND IMMUNOTHERAPY

The same regimens are important in the metastatic setting, with cisplatin-based therapy as the first-line choice. Immunotherapy is the preferred second-line treatment, but can be given first-line when patients are not cisplatin candidates (creatinine clearance < 60 mL/min).

Five checkpoint inhibitors have been approved for metastatic bladder cancer: atezolizumab (Tecentriq), nivolumab (Opdivo), avelumab (Bavencio), durvalumab (Imfinzi), and pembrolizumab (Keytruda), all of which have a similar mechanism of action. Dr. Shah cited data for several of these agents, all used in the second-line setting.

Atezolizumab was approved in 2016 based on a single-arm study in which patients with locally advanced or metastatic disease had a median overall survival of 11.4 months, vs. 6.5 and 6.7 months in the control arms; 12-month survival was 48%, 30%, and 29%, respectively (Rosenberg et al., 2016). A more recent phase III study, however, reportedly did not meet its primary endpoint of overall survival, although further details were unavailable at the time of the presentation.

Nivolumab, in a single-arm phase II trial, produced responses in 19.6% of patients, leading to a median overall survival of almost 9 months (Sharma et al., 2017). In KEYNOTE-045, pembrolizumab compared to chemotherapy reduced the risk of death by 27% (*p* = .002), based on a median overall survival of 10.3 months, vs. 7.4 months with chemotherapy, although progression-free survival was similar between the arms (Bellmunt et al., 2017).

In these key studies, patients with high expression of programmed cell death ligand 1 (PD-L1) had somewhat higher response rates than PD-L1–negative patients, although all-comers benefited as compared to traditional treatment.

"Based on this level 1 evidence, pembrolizumab has the highest recommendation for platinum-refractory patients or platinum-eligible patients," she said.

## MANAGEMENT OF IMMUNOTHERAPY-RELATED TOXICITIES

Practitioners at MD Anderson have a standard set of laboratory parameters they check before starting patients on immunotherapies (Table 1).

**Table 1:**
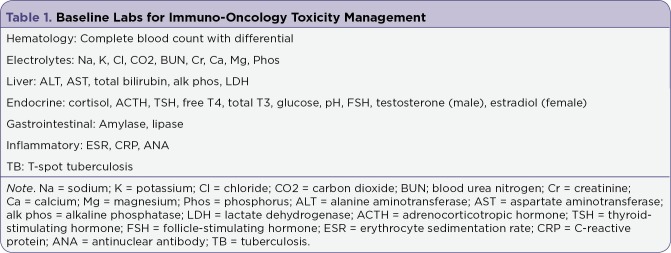
Baseline Labs for Immuno-Oncology Toxicity Management

"Having those baseline studies helps us tease out underlying causes, when we see conditions arise that may be side effects related to immunotherapy," Dr. Shah said. "It is important to tell patients that most people will tolerate immunotherapy very well, but 10% have significant side effects. Educate your patients on the most common ones, and emphasize that they need to call you if anything seems off."

The main side effects are endocrinopathies, diarrhea and colitis, pneumonitis, and myositis. A number of tests can be employed to confirm these. Early detection is key, and the mainstay of treatment is corticosteroids (1 mg/kg twice per day). Steroid-refractory patients with colitis can often be helped by mesalamine or budesonide.

## AREAS OF EXPLORATION

According to Dr. Lemke, many questions are yet to be answered regarding immunotherapy in urothelial cancer, including the utility of PD-L1 expression in selecting patients, the relative superiority of PD-1 vs. PD-L1 blockade, the benefit of combining anti–PD-1/PD-L1 agents with ipilimumab (Yervoy) or with cytotoxic chemotherapy, the optimal sequencing of treatments, the duration of treatment, and the role of immunotherapy in the curative-intent setting.

Another active area of research is the molecular characterization of bladder cancer. So far, three main subtypes have emerged:

- Basal: aggressive biology, highest proliferation markers, poorer outcomes, chemotherapy-sensitive
- Luminal: predominant in micropapillary tumors, mutations in fibroblast growth factor receptor 3 (*FGFR3*) are common, initially superficial but progress to muscle-invasive
- P53-like: Low proliferation, stromal enrichment, chemotherapy-resistant, bone-trophic, better outcomes.

"Molecular characterization will be a key principle in bladder cancer in the years to come, and will help guide our treatments," she said.
